# A Comprehensive Peptidomic Approach to Characterize the Protein Profile of Selected Durum Wheat Genotypes: Implication for Coeliac Disease and Wheat Allergy

**DOI:** 10.3390/nu11102321

**Published:** 2019-10-01

**Authors:** Rosa Pilolli, Agata Gadaleta, Luigia Di Stasio, Antonella Lamonaca, Elisabetta De Angelis, Domenica Nigro, Maria De Angelis, Gianfranco Mamone, Linda Monaci

**Affiliations:** 1 Institute of Sciences of Food Production, CNR-ISPA, 70126 Bari, Italylinda.monaci@ispa.cnr.it (L.M.); 2 DiSAAT, Università degli Studi di Bari Aldo Moro, 70126 Bari, Italy; 3 Institute of Food Sciences, CNR-ISA, 83100 Avellino, Italy; 4 DiSSPA, Università degli Studi di Bari Aldo Moro, 70126 Bari, Italy

**Keywords:** in-vitro gastroduodenal digestion, celiac disease, wheat allergy, epitopes, peptidomic approach, high resolution mass spectrometry

## Abstract

The wheat varietal selection undertaken by breeders in recent decades has been tailored mainly to improve technological and productivity-related traits; however, the latter has resulted in a considerable impoverishment of the genetic diversity of wheat-based products available on the market. This pitfall has encouraged researchers to revalue the natural diversity of cultivated and non-cultivated wheat genotypes in light of their different toxic/immunogenic potential for celiac disease and wheat-allergic patients. In the present investigation, an advanced proteomic approach was designed for the global characterization of the protein profile of selected tetraploid wheat genotypes (*Triticum turgidum*). The approach combined proteins/peptides sequence information retrieved by specific enzymatic digestions (single and dual proteolytic enzymes) with protein digestibility information disclosed by means of in-vitro simulated human gastroduodenal digestion experiments. In both cases, the peptide pools were characterized by discovery analysis with liquid chromatography high-resolution tandem mass spectrometry, and specific amino acid sequences were identified via commercial software. The peptide list was screened for in silico toxicity/immunogenicity risk assessment, with the aid of various open-source bioinformatics tools for epitopes matching. Given the global information provided by the designed proteomic approach, the in silico risk assessment not only tackled toxicity implication for celiac disease patients, but also scouted for immunogenic sequences relevant for wheat allergic patients, achieving a comprehensive characterization of the protein profile of the selected genotypes. These latter were assessed to encrypt a variable number of toxic/immunogenic epitopes for celiac disease and wheat allergy, and as such they could represent convenient bases for breeding practices and for the development of new detoxification strategies.

## 1. Introduction

Wheat is one of the most important and widely consumed cereals in the world, and it is the preferred choice for bread and pasta making, due to its peculiar technological properties. The wheat proteins consist of two main classes: (i) albumin/globulin and (ii) storage proteins [1]. The albumin/globulin proteins account for about the 20% of the total grain proteins. They include water and salt-soluble proteins and are mainly represented by metabolic, regulatory and protective enzymes involved in important functions related to the development of the plant and responses to the environment [2,3].

The storage proteins are represented by gluten proteins, which are classified as monomeric, alcohol-soluble prolamins (gliadins) and polymeric, alcohol-insoluble glutelins (glutenins). Gliadins are divided into subgroups α, β, γ, and ω according to their electrophoretic mobility in acid PAGE, while glutenin subunits are classified as either high molecular weight (HMW-GS, type-x, or type-y) or low molecular weight (LMW-GS, s-, m- and i-type) [4].

In addition to their functional and rheological properties, wheat proteins may activate several inflammatory responses in susceptible individuals, with different immunological pathways, such as celiac disease (CD), atopic dermatitis, urticarial and wheat allergies (WA), i.e., baker’s asthma, and wheat-dependent exercise-induced anaphylaxis (WDEIA) [5]. Celiac disease is an autoimmune enteropathy triggered by the ingestion of gluten proteins in genetically susceptible individuals which express at least one of the human leukocyte antigen (HLA) genes DQ2 and DQ8. The consumption of gluten in CD patients causes self-perpetuating damage to the intestinal mucosa, leading to a malabsorption of nutrients. The α-gliadins are most frequently associated with CD; however, several toxic/immunogenic epitopes derive also from γ-gliadins, ω-gliadins, HMW-GS, and LMW-GS [6,7]. The main allergens associated with WDEIA are ω-gliadins and HMW-GS, but LMW-GS can also trigger WDEIA due to a partial homology in the amino acid sequences with the major allergen ω5-gliadin [8,9,10]. In addition, water/salt-soluble proteins, such as α-amylase/trypsin inhibitors, β-amylase, peroxidases, serpins, and α-purothionin, have also been reported to trigger IgE-mediated allergies related to wheat ingestion [11].

The intrinsic genetic diversity and the considerable allelic variation among different cultivars account for a high heterogeneity in the primary structure of wheat proteins (in particular, the storage proteins) generated by amino acid insertions, deletions and substitutions. Notably, the overall complexity of the wheat proteomic profile may disclose a differential immunostimulatory potential, as well as a differential susceptibility to the enzymatic degradation in the gastrointestinal tract, among different genotypes (species, cultivars and breeding lines) [12]. The varietal selection undertaken by breeders in the last decades, mainly tailored towards improving technological and productivity related traits, has caused, as a main drawback, a considerable reduction in diversity among commercially available wheat cultivars. Starting from this, researchers are being encouraged to revalue the natural diversity of cultivated and non-cultivated wheat genotypes, tracing back its effect on the differential toxic/immunogenic potential for celiac disease and wheat-allergic patients [13].

In a recent investigation, we presented a detailed characterization of a tetraploid wheat collection containing 38 accessions of durum wheat (*Triticum turgidum*) [14] selected from a wider list of 240 genotypes, developed at the University of Bari Aldo Moro, including both wild and cultivated accessions [15]. The collection was investigated using a multidisciplinary approach including conventional proteomic profiling focused on the gliadin fraction (HPLC-UV and R5-ELISA), yield and quality traits of the whole grains (grain yield per spike, grain total protein content, dry gluten and gluten index) [14]. A statistical evaluation of the acquired data allowed the identification of a short list of candidate genotypes, combining reduced gluten content with satisfactory rheological properties required for their perspective usability in bread or pasta [14]. The five selected genotypes were subjected to in vitro simulated human gastroduodenal digestion experiments and untargeted HR-MS/MS analysis in order to investigate the protein digestibility and the potential toxicity for celiac disease patients. Interestingly, the number of intact immune-active peptides detected in all the five genotypes under investigation was lower than in the reference sample of commercial semolina, and this quite promising result demanded further detailed investigation.

In accordance with this input, in the present work we carry out an in-depth analysis of the proteomic profile of the wheat genotypes previously selected. An advanced proteomic approach was carried out combining the information provided by: (i) the comprehensive protein identification of protein fractions (albumin/globulin and storage proteins); and by (ii) the identification of gastroduodenal resistant peptides which survive the proteolytic activity of digestive enzymes and, as such, pose a risk for celiac disease and wheat allergy patients. The latter was applied directly to raw flours, according to the standardized static protocol proposed by Minekus et al. in 2014 [16]. The peptide pools generated in all the steps were analyzed by liquid chromatography high resolution tandem mass spectrometry in data dependent^TM^ acquisition mode with an instrumental method tailored to widen the amount of information retrieved and a dual-round software-based sequence identification. The full list of enzyme-specific peptides and gastroduodenal resistant peptides were filtered according to specific criteria of reliability for the highest confidence in sequence identification, and finally, the refined list was screened for in silico toxicity/immunogenicity risk assessment in order to tackle implications for celiac disease and wheat allergy.

## 2. Materials and Methods

### 2.1. Plant Materials

The genotypes were grown in the experimental field “A. Martucci” of the Department of Soil, Plant and Food Sciences at Valenzano (Bari, Italy) in 2015, in a randomized complete block design. Full details about agronomic practices were described elsewhere [14].

### 2.2. Sequential Protein Extraction of Osborne Fractions and Total Protein Quantification

Sequential protein extraction was carried out on 100 mg of non-defatted flours as previously described with few modifications [17]: (i) water-soluble protein extraction was performed by a buffered salt solution (2 × 1 mL 0.067 M phosphate buffer, 0.4 M NaCl, pH 7.6) at room temperature to separate albumins and globulins (F1-ALB/GLO); (ii) the gliadin fractions were collected with alcoholic solution (3 × 0.5 mL 60% ethanol solution) at room temperature (F2-GLIA); (iii) finally the glutenin fractions (F3-GLU) were extracted with an alcoholic, denaturing and reducing solution (2 × 1 mL 50% 1-propanol solution containing 0.05 M TrisHCl, 2 M urea and 1% DTT at pH 7.5). After the addition of each extraction solution, the suspensions were vortexed for 2 min, and shaken for 10 min at room temperature (as for F1-ALB/GLO and F2-GLIA) or shaken for 20 min at 60 °C (as for F3-GLU) (KS 4000 i-control shaker, IKA Works GmbH &Co. KG, Staufen, Germany). The supernatants from each step were collected after centrifugation (10 min at 5000 rcf).

The total protein content of the three fractions was quantified by a commercial kit based on the Bradford colorimetric assay (Biorad srl, Milan, Italy), according to the producer instructions. Statistical differences in protein content were determined by multiple *t*-tests comparing one-by-one the mean values of the reference sample with each genotype at a significance level of 5% (hypothesis case: comparison of small samples with unknown but equal variances, the latter assumption was taken after a proper F-test for equality of two variances).

### 2.3. Bottom-Up Approach for Protein Characterization

#### 2.3.1. Single Enzyme Digestion

Aliquots of the protein fractions were diluted in the digestion buffer suggested by the producer for optimal chymotrypsin activity (0.1 M TrisHCl, pH 8 + 10 mmol/L CaCl_2_) to a final volume of 200 µL. The dilution factor was different based on the original protein concentration, in particular, the samples F1-ALB/GLO were diluted 1:10, the samples F2-GLIA were diluted 1:10, and the samples F3-GLU were diluted 1:5. Before digestion, protein denaturation (15 min at 95 °C), reduction (incubation for 30 min at 60 °C after addition of 10 µL of 50 mmol/L dithiothreitol solution) and alkylation were performed (incubation for 30 min at room temperature after addition of 20 µL of 100 mmol/L iodacetamide solution). Finally, a proper amount of chymotrypsin solution (0.25 µg/µL in HCl 1 mmol/L) was added to each sample in order to provide an enzyme-to-protein ratio at least equal to 1:20. The samples were incubated for 16 h under shaking at 25 °C and the reaction was stopped by acidifying the sample with 2 µL of 6 mol/L HCl. The samples obtained according to this procedure were labeled as CHY digests.

#### 2.3.2. Dual Enzyme Digestion

Aliquots of the protein fractions were diluted in the digestion buffer suggested for thermolysin by the producer (0.05 M TrisHCl, pH 8 + 0.5 mol/L CaCl_2_) to a final volume of 200 µL. The samples F1-ALB/GLO were diluted 1:10, the samples F2-GLIA were diluted 1:20, and the samples F3-GLU were diluted 1:5. Before digestion, protein denaturation, reduction and alkylation were performed like detailed in the previous paragraph. The first digestion step was carried out by incubation with thermolysin for 4 h at 95 °C under shaking. 4 µL of thermolysin solution 0.5 µg/µL was added in order to achieve an enzyme/protein ratio higher than 1:20. After cooling down at room temperature the same samples were subjected to a second enzymatic digestion step by addition of a proper amount of chymotrypsin solution 0.25 µg/µL (enzyme-to-protein ratio higher than 1:20). The reaction was stopped by acidifying the sample with 2 µL of 6 mol/L HCl. The samples obtained according to this procedure were labeled as THE/CHY digests.

### 2.4. SDS-PAGE Analysis

20 µg of protein (F1-ALB/GLO, F2-GLIA, F3-GLU) was separated, under reducing conditions, by means of sodium dodecyl sulfate-polyacrylamide gel electrophoresis (SDS-PAGE) on polyacrylamide pre-cast gels (8.6 cm × 6.7 cm × 1 mm) using a Mini-Protean Tetra Cell equipment (Biorad srl, Milan, Italy). 4–20% gels were used for separation of samples F1-ALB/GLO, F2-GLIA, whereas a 12% gel was used for F3-GLU. Samples were diluted 1:1 in Laemmli buffer and denatured for 5 min at 95 °C. Further experimental details were described elsewhere [18].

### 2.5. In Vitro-Simulated Human Gastroduodenal Digestion Experiments

Wheat flours (1 g) were subjected to in-vitro simulated human gastroduodenal (GD) digestion according to the standardized static model proposed by Minekus et al. in 2014 [16]. Details about GD digests preparation and purification were reported elsewhere [14,19].

### 2.6. Discovery HR-MS/MS Analysis and Peptide Identification

Micro-HPLC-MS/MS analyses were performed on an Ultimate 3000 UHPLC system coupled to a hybrid quadrupole-Orbitrap^TM^ mass spectrometer Q-Exactive Plus (Thermo Fisher Scientific, San Josè, CA, USA). Peptide separation was accomplished on an Acclaim PepMap100, C18 column, (3 μm, 100 Å, 1 × 150 mm) at a flow rate of 60 µL/min and the untargeted high-resolution MS/MS analysis was performed by Full-MS/dd-MS2 analysis mode. All the instrumental set-up was described previously [14]). Raw data were processed by Proteome Discoverer v.2.1 sp1 (Thermo Fisher Scientific) for peptide/protein identification. The Sequest HT searching algorithm was applied against customized databases with the following features:

CHY and THE/CHY digests. Database downloaded from Uniprot [20] on 6 March 2019, including taxonomy Poaceae ID 4479 (total 1.8 million accessions). As for CHY digests, specific cleavage was predicted at FLWY residues. As for THE/CHY digests, a combined specificity was set up including the cleavage sites of both enzymes (AFILMVWY). The processing workflow was set as follows: mass tolerance on the precursor and fragment ions 5 ppm and 0.02 Da, respectively; maximum 5 missed cleavage; peptide length 4–144 amino acids (AA); dynamic modifications: methionine-oxidation, asparagine/glutamine-deamidation, n-terminal glutamine cyclization to pyroglutamate, N-terminal protein acetylation; static modification: cysteine carbamidomethylation. 

GD digests. Database downloaded from Uniprot [20] on 22 March 2019, including taxonomy Triticum ID 4564 (179000 accessions). A non-specific cleavage was set for the .fasta file index. The processing workflow was set as follows: mass tolerance on the precursor and fragment ions 10 ppm and 0.02 Da, respectively, 0 missed cleavage, peptide length 4–144 amino acids (AA), dynamic modifications: methionine-oxidation, asparagine/glutamine-deamidation, n-terminal glutamine cyclization to pyroglutamate, N-terminal protein acetylation.

Specific filters on the software output were applied in order to constrain the protein/peptide lists to the most reliable sequence identifications. Protein filters: at least 1 unique peptide, score Sequest HT ≥ 1, number of peptide spectrum matches (PSMs) ≥3, confidence level at least medium (FDR ≤ 5%). Peptide filters: unambiguous PSMs, XCorr Sequest HT ≥1, number of PSMs ≥3, confidence level at least medium (FDR ≤ 5%).

### 2.7. In Silico Toxicity/Immunogenicity Risk Assessment

The occurrence of linear epitopic sequences relevant for celiac disease patients was assessed by means of the CD Novel Protein Risk Assessment Tool, which is provided open source by the FARRP at University of Nebraska, (CELIAC Database, Beta-3 Release) [21].

The occurrence of linear epitopic sequences relevant for wheat allergic patients was assessed by querying the IEDB database (https://www.iedb.org/) [22]. The following filters were applied for the bioinformatic search: linear epitope (substring for BLAST), host humans, positive assay only, any MHC restriction, allergic disease.

## 3. Results and Discussion

The main objective of this work is the comprehensive characterization of the proteomic profile of five wheat genotypes, which were selected in our previous investigation [14]. The main information regarding such genotypes is summarized in Table 1.

The grains were collected from plants grown under consistent conditions in a field-trial at the same location during the same growing season, therefore, the influence of environmental factors on the protein expression was neglected, and any difference was ascribed to the genetic diversity. The proteomic profile of the selected genotypes will be compared with commercial semolina samples, namely, products that are actually included in the current human diet. Most of the commercial samples, unless differently labeled, are derived from a mixture of different cultivar/genotypes, usually not specified, selected only for their productivity and technological traits. A recent debate is that the genetic improvement of wheat may have led to greater immunogenicity and, consequently, to a higher prevalence of celiac disease/wheat allergy [23]. Therefore, we chose as reference a commercial sample of durum wheat semolina purchased directly from the market, which as “multivarietal mixture”, has a good chance of being representative of modern commercial varieties. The comparison with a wheat flour grown under different condition might induce a bias in the final observed differences due to the effect of environmental conditions and agronomic practice on the protein expression level. However, given the hundreds of genotypes cultivated worldwide and the lack of general indications about reliable reference sample for this kind of comparative investigation, we deemed this choice to be the best feasible option.

**Figure 1:**
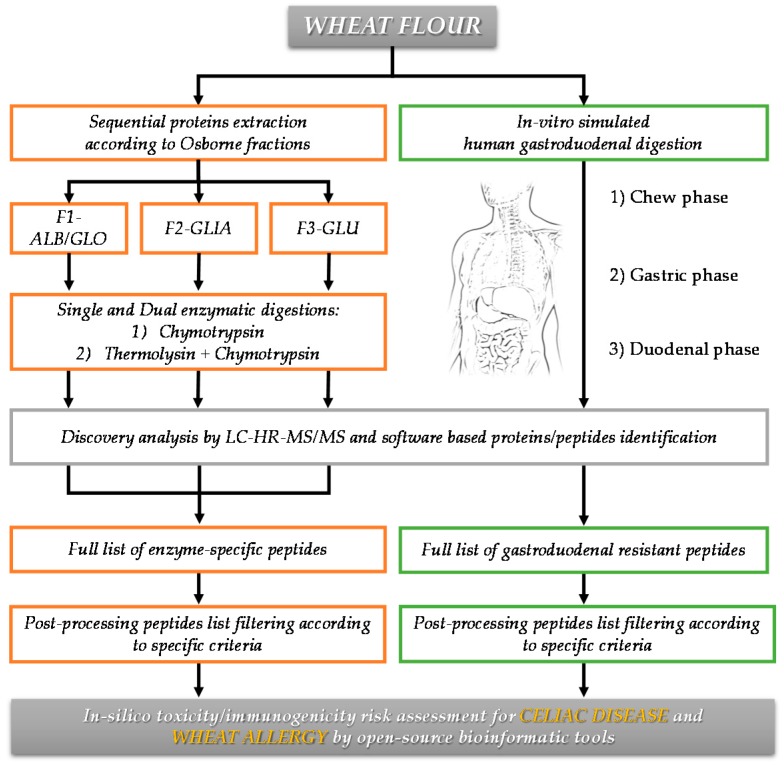
Description of the analytical workflow applied for the comprehensive peptidomic approach to characterize the protein profile of wheat genotypes.

The genotypes listed in Table 1 were selected for their satisfactory rheological properties, combined with reduced gluten content (R5-ELISA immune reactivity); moreover, tracking the fate of gluten protein upon human gastroduodenal digestion experiments, they all disclosed a significantly lower number of toxic epitopes than the commercial sample of semolina [14]. These promising preliminary results raised the need for a detailed proteomic investigation in order to characterize the whole proteomic profile and retrieve more information about their immunogenic/toxic potential. With this aim, we designed the analytical workflow schematized in Figure 1. The approach combined peptide sequence information provided by (i) specific enzymatic digestions (single and dual proteolytic enzymes) with protein digestibility information disclosed by means of (ii) in vitro simulated human gastroduodenal digestion experiments. Both routes were accomplished on raw wheat flours of each genotype. Details about methodology and main results will be discussed in the following sections.

### 3.1. Specific Enzymatic Digestion of Osborne Protein Fractions

Sequential protein extraction of albumin/globulin and storage proteins (gliadin and glutenin) was carried out for each genotype, according to the different protein solubility in aqueous or alcoholic solutions. The three fractions, labeled as F1-ALB/GLO, F2-GLIA and F3-GLU, were collected, and their total protein content was quantified by means of a commercial colorimetric Bradford assay in order to compare the extracted protein amount for each genotype with the reference sample of commercial semolina (see Figure S1). One-by-one comparisons by *t*-test were carried out between each genotype and the reference sample, and statistically significant differences were highlighted with asterisks.

Interestingly, all five genotypes presented a higher protein concentration than the reference semolina in the first fraction (F1-ALB/GLO), which should preferentially contain water/salt soluble proteins generally identified as globulins and albumins, whereas non-significant differences were disclosed for the gliadin fractions (F2-GLIA), except for sample 5 (lower concentration). As for the third fraction (F3-GLU), a lower amount of total glutenin subunits in the genotypes 1, 3 and 5 was detected compared to the reference semolina.

Focusing on potential difference in the primary protein structure, the collected fractions were subjected to specific enzymatic digestion and discovery high resolution tandem mass spectrometry (HR-MS/MS) analysis in order to identify the protein amino acid sequences. Given the low amount of basic residues present in wheat storage proteins, we selected valid alternatives to the most common trypsin as specific cleavage enzyme. In particular, chymotrypsin (CHY) was selected as the main enzyme and, in order to widen the information retrieved, we also carried out a dual enzyme digestion protocol combining the proteolytic activity of thermolysin and chymotrypsin (THE/CHY).

The peptide pools obtained by these experiments were analyzed by untargeted HR-MS/MS analysis, and the raw data processed by commercial software designed for sequence identification. The Sequest HT algorithm was applied for searches against a customized database, containing all the known accessions of the *Poacee* taxonomy [20]. Wide constraints were set for sequence identification, the final aim being to keep the software-based search as comprehensive as possible, while critically evaluating the software output afterwards. In addition, in order to maximize the protein coverage, we decided to widen the maximum limit of missed cleavage to five, even if this increased the processing time, as well, due to the specific properties of gluten proteins, which are particularly resistant to the enzymatic proteolytic activity. With a preliminary test, we compared the protein coverage achieved via software by applying a maximum of either 2 or 5 missed cleavages in the processing of the same data set, and we find out some benefit from the latter option, in terms of protein coverage.

The identification study via Proteome Discoverer (PD) was designed with specific categorical factors related to the three protein fractions (either F1 or F2 or F3), and the enzymatic cleavage carried out (either CHY of THE/CHY), to maintain information about the analytical sample. Still, a unique multiconsensus protein/peptide list was generated for each genotype in order to provide a maximum coverage of the protein sequences. The “multiconsensus” was achieved by a specific option of the commercial software used for the protein identification. It is an automatic procedure, which makes it possible to process spectra from different chromatographic runs collating multiple information in a unique result file, without redundancy of information.

**Figure 2:**
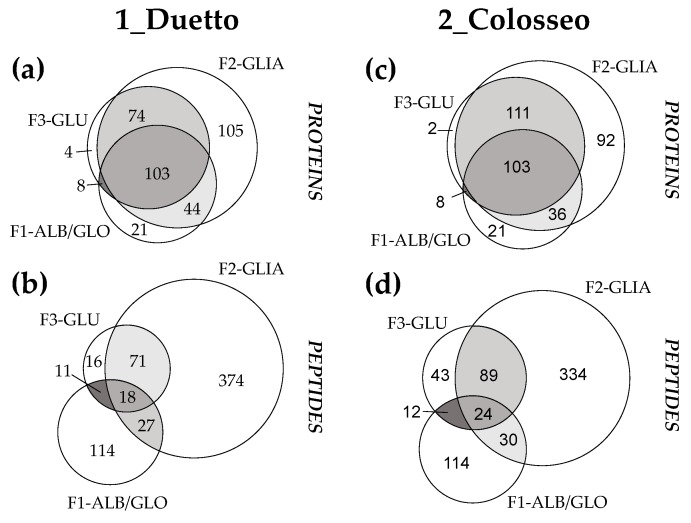
Venn diagrams highlighting the distribution of identified proteins (panels (**a**) and (**c**)) and peptides (panels (**b**) and (**d**)) among the three different protein fractions subjected to specific digestion with chymotrypsin.

The final protein/peptide lists were filtered according to specific criteria detailed in Section 2.6, for high confidence in sequence identification. Notably, the Venn-diagrams reported in Figure 2 highlight the importance of analyzing all three sequential fractions for each genotype in order to achieve a comprehensive characterization of the proteome and peptidome. 

As an example, the distribution of identified proteins (panels (a) and (c)) and peptides (panels (b) and (d)) among the three different fractions were displayed for wheat varieties labeled as 1 and 2, but the same general trend was highlighted for the other samples. Some protein accessions and most of the peptide sequences were uniquely identified in a specific fraction; however, some others were actually shared among the fractions. Significant overlapping was observed between the fractions F2 and F3, related to storage proteins, and this evidence was ascribed to two main reasons: (i) the partial extraction of some glutenin subunits already in the ethanolic fraction; (ii) the sequence homology between LMW-GS and gliadins, which affects the proper peptide/protein identification [24].

In Table 2, we summarized the main features obtained by discovery analysis in terms of total number of protein and peptide for each sample. Notably, the chymotriptic enzymatic digestion itself already provided quite a wide range of information about protein and peptide compositions of the selected genotypes (see the first two lines of Table 2). However, by implementing the dual enzyme digestion THE/CHY, and combining these results with the previous round of identification, we were able to increase the number of both proteins and peptides detected for each genotype, achieving a final protein coverage higher than 25% for about 40% of the identified proteins. The full list of matched peptides and relevant protein accessions can be found in the Supplemental Information (Table S1). 

As for storage proteins, the hundreds of protein accessions identified can actually be restricted to a short list of protein groups, due to both redundancy of information in the Uniprot database and to the presence of wide repetitive motifs in the primary structures of gliadin and glutenin proteins. In summary, all categories of gliadins and glutenin were detected: α/β-gliadins (total of 138 accessions), γ-gliadins (total of 118 accessions), ω-gliadins (ω_5_ and ω_1,2_, total of 61 accessions), HMW-GS (x-type and y-type, total of 42 accessions) and LMW-GS (s-type, m-type and i-type, total of 175 accessions). As for albumin/globulin proteins, several accessions were detected as well, belonging to globulins (3A, 1 and 1S), α–amylase inhibitors (CM16, CM2, CMX1/CMX3, CM3, 0.28, 0.19), non-specific lipid transfer proteins (ns-LTP), α-/β-purothionin, serpins (Z1C, Z1A and 3), peroxidase, glyceraldehyde 3-phosphate dehydrogenate and several others minor proteins. Notably, most of the detected proteins present toxic/immunogenic potential for CD and WA patients.

Afterwards, each fraction was subjected to monodimensional electrophoretic separation by SDS-PAGE for protein profile qualitative characterization. In Figure S2, we report the typical SDS-PAGE profiles obtained. The tentative band attribution was accomplished according to the list of proteins identified by the previous bottom-up investigation and relevant molecular weights (MWs).

### 3.2. In Vitro-Simulated Human Gastroduodenal Digestion Experiments

The wheat flours were subjected to in vitro simulated human gastroduodenal digestion (GDD) experiments. The standardized static method proposed by Minekus et al. in 2014 [16] and developed within the COST Infogest network was applied for physiological conditions. The cited protocol provides specific recipes for the three digestion phases, namely, oral, gastric and duodenal, setting several parameters such as buffer compositions, matrix/enzyme ratios, incubation conditions, etc. For this experiment, we considered only the analytical readout of the duodenal endpoint in order to assess the presence of GD resistant peptides persistent at the end of the duodenal phase, which might pose immunogenicity/toxicity risk in susceptible individuals. After a purification by solid phase extraction, the GD digests were analyzed by untargeted HR-MS/MS analysis.

Due to the high complexity expected for this set of samples, and aiming at a comprehensive peptidomic characterization, we devised a two-round identification protocol exploiting specific features of the MS instrumental platform.

**Figure 3:**
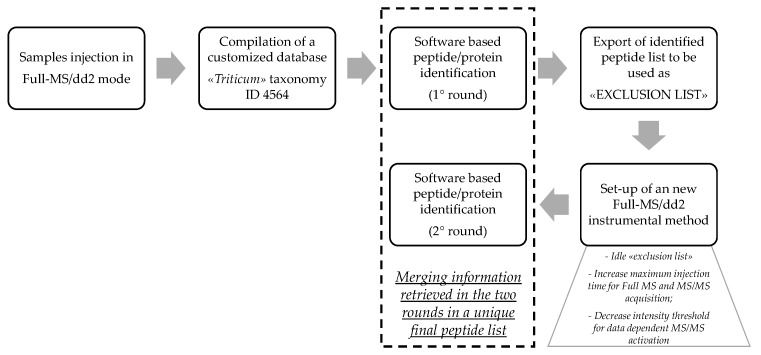
Workflow of the two-round identification protocol applied to maximize the number of GD resistant peptides identified.

The workflow is described in Figure 3. Briefly, we performed a first round of discovery analysis in which the instrumental set-up was based on a common data dependent^TM^ template for untargeted peptide analysis, the same one was applied for characterization of enzyme specific digests of Osborne fractions. The MS/MS spectra collected were processed via PD by setting the general parameters itemized in Section 2.6. In this case, we set a wide mass accuracy (10 ppm), due to the expected matrix complexity being higher than the Osborne fractions. Notably, given the high heterogeneity and low specificity of the proteolytic cleavage occurring in the different GD digestion phases, we applied the ‘unspecific’ cleavage option for database indexing. With this option, the software automatically calculates all the possible hydrolyzed peptides in any available site within the mass range set for the indexing. This raises exponentially the processing time and the amount of data generated; therefore, we selected for this experiment a restricted database containing only the *Triticum* taxonomy (ID 4564). Theoretically, using different databases for protein/peptide identification can cause bias in the final list of accessions and peptide sequences detected. To exclude this drawback, we carefully screened the final list of protein accessions detected in the CHY and THE/CHY digests with the *Poaceae* database and we assessed that all the top-scored proteins belonged to either the *Triticum* taxonomy or the *Aegilops*, the latter being diploid ancestor of hexaploid wheat.

This first round of identification provided a preliminary list of peptide sequences which were exported from the PD software and uploaded in the MS method file to work as exclusion list. In addition, the acquisition file was also slightly modified in terms of maximum injection time (increased), and intensity threshold to activate MS/MS fragmentation (decreased), in order to increase the overall sensitivity of the analysis. With these new features, new acquisitions of the same samples were carried out and the second set of MS/MS spectra was processed by PD with the same search parameters. Finally, a unique consensus list of peptide sequences was compiled for further investigation. Like before, the list of detected peptides were filtered according to specific reliability criteria for the highest confidence in identification (see Section 2.6 for details) and the final outcome is reported in Table S2 of the Supplementary Information. In summary, we identified a total of 3010 gastroduodenal resistant peptides for the reference sample of commercial semolina, and 2178, 2677, 2515, 2491 and 2408 for the five selected wheat genotypes from sample 1 to 5, respectively. This analytical approach made it possible to outperform our own preliminary investigation, accomplished in the recently published paper [14]. Notwithstanding the wider information retrieved, the identified GD resistant peptides were mostly assigned to storage proteins, whereas only a few fragments belonged to albumin/globulin proteins, in particular, β-amylase, α-amylase/trypsin inhibitors, ns-LTP, peroxidase, serpins. This experimental evidence could be ascribed not only to the lower concentration of the metabolic proteins, which accounts for only 20% of the total seed proteins, but also to their differential susceptibility to the proteolytic degradation by human digestive enzymes. Indeed, focusing on the main water/salt-soluble proteins detected in the GD digests, it was noticed that a low protein coverage was achieved (below 40%), with many short fragments below 7 AA in length. According also to previous findings reported by Mamone et al. 2015 [25], we can conclude that ns-LTP and the α-amylase/trypsin inhibitors both exhibited high resistance to proteases; therefore, they were mainly undigested in the final GD sample. Whereas, other components of the albumin/globulin fraction likely underwent complete degradation by digestive proteases [25].

### 3.3. In Silico Toxicity/Immunogenicity Risk Assessment and Implication for Celiac Disease and Wheat Allergy Patients

The comprehensive information provided by the designed peptidomic approach served the final aim of evaluating in silico the toxicity/immunogenicity risk of these genotypes in susceptible individuals [26]. Given the global characterization involving both the albumin/globulin and the storage proteins, the risk assessment was carried out not only tracing potential toxicity for celiac disease patients, but also scouting for immunogenic sequences relevant for wheat-allergic patients. Various open-source bioinformatics tools were used for epitopes matching.

For CD epitopes searches, the CELIAC Database v.2 and the relevant tool for protein risk assessment was used to identify exact matches between the detected GD resistant peptides and known immune-stimulatory sequences. First the enzyme-specific peptide list obtained by CHY and THE/CHY digestion of Osborne protein fractions was screened. Several full-length epitopes were detected, belonging to the main storage proteins, namely, α-gliadins and γ-gliadins but also ω-gliadins and glutenin (both low and high molecular weight subunits). The complete list of CD epitopes was appended as Table S3, with all the details about identification number, full length sequence, HLA-DQ binding protein, core epitope, and, finally, the wheat genotype they belonged to.

Similarly, the search of CD intact epitope was carried out on GD resistant peptides. In this case, the list of identified peptides was first filtered with a size cut-off of 9AA in order to investigate only peptides with T-cell stimulatory potential. Indeed, fragments shorter than 9 AA would be unable to bind to MHC class II molecules and to activate T-cells [27]. In Table 3 (lines 1 and 2), we show the results of this qualitative investigation, reporting the total number of identified GD resistant peptides for each genotype and the relevant subset recognized as a safety hazard for full match with T-cell stimulatory epitopes. Interestingly, the number of peptides encrypting CD epitopes was significantly reduced for all the genotypes under investigation, but particularly low for the genotype number 5 (PI 56263), which encoded less than the 30% of hazard peptides compared to the reference sample of semolina. This new experimental evidence was very promising, and actually confirmed the general trend disclosed in our previous preliminary work [14]. The comprehensive peptidomic investigation supplied a wider information about the selected genotypes; in addition, the dual-round identification approach applied to characterize the GD digests provided more confidence in the final results, due to the significant number of detected peptides that were screened in silico for their toxicity risk.

**Figure 4:**
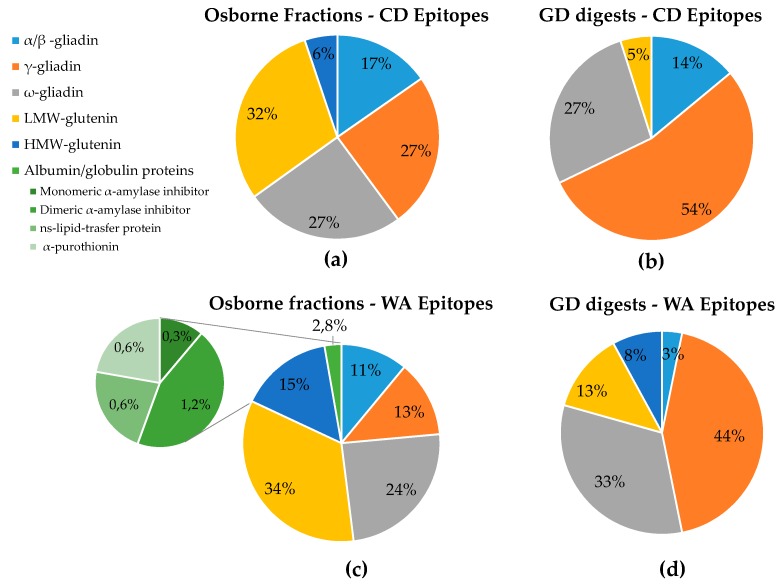
Distribution among main proteins of the detected CD (**a**,**b**) and WA (**c**,**d**) epitopes in Osborne fractions and GD digests.

Figure 4 provides a general map of the hazard peptides distribution among different wheat proteins. In panels (a) and (c), we pooled all spectra acquired for the three fractions with both the CHY and THE/CHY enzymes and their technical replicates. The pie chart proves that the devised analytical approach works properly, since it is able to characterize all the main wheat proteins in terms of immunogenic/toxic epitopes occurrence. Similarly, Figure 4 panels (b) and (d) relies on collated data from the GD digests spectra, merging all technical replicates of the wheat genotypes under investigation. 

As for CD, the epitopes belonged only to storage proteins (see panels (a) and (b)). The distribution of epitope-containing peptides was quite homogeneous for the discovery analysis accomplished on Osborne fractions (Figure 4a), ranging between a minimum of 17% for α-gliadins and a maximum of 32% for LMW-GSs; in contrast, HMW-GS epitopic sequences accounted for only 6% of the total hazard sequences. Notably, when performing the same analysis on GD digests, the final distribution among storage proteins changed dramatically. Indeed, all the HMW-GS and most of the LMW-GS epitopic sequences were not detected at the end of the duodenal digestion phase, and α-gliadins and ω-gliadins persisted almost unchanged, while most of the hazard peptides belonged to γ-gliadins (see Figure 4b). These results support the hypothesis of a differential resistance of the aforementioned proteins to the hydrolytic activity of digestive enzyme. Notwithstanding their higher toxicity, the α-gliadins were proved to be more susceptible to proteolytic degradation by pancreatic enzymes than the γ-gliadins. Indeed, they are almost completely hydrolyzed, except for the main immunogenic peptides [28].

**Figure 5:**
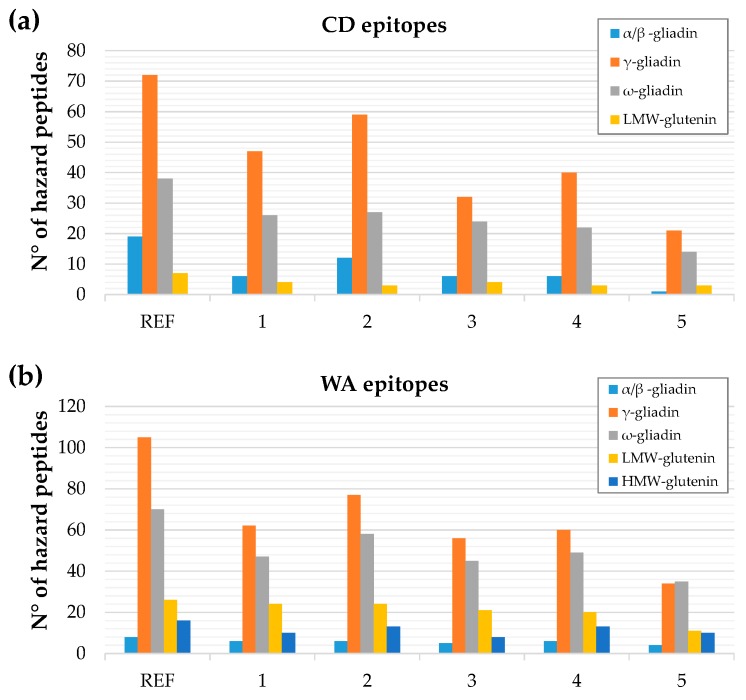
Distribution among the main proteins of the hazard peptides detected in GD digest containing intact epitopes relevant for CD (**a**) and WA (**b**).

Focusing on the CD epitopic peptides distribution among different genotypes and specific proteins, we reported in Figure 5a the general trend observed in GD digests. Interestingly, the selected lines coded for a reduced number of hazard peptides for all gliadin and glutenin types, but particularly for the α-gliadins. Due to the high sequence homology between γ- and ω-gliadins, the count of hazard peptides might be slightly overestimated for these two categories.

In Table S3, the information provided by the list of hazard peptides was handled in term of specific sub-types of CD full length epitopes detected. The sequences were itemized according to the identification number assigned by the CELIAC Database [19] and, whenever possible, the 9 AA core epitopes, with relevant standardized nomenclature [7] was reported, in order to collate information about repetitive motifs. Through alignment of the data collected for the Osborne fractions and the GD digests, a differential susceptibility of the detected sequences to the digestive enzymes was assessed. Indeed, all of the epitopes containing the cores DQ8-glia-α1, DQ2.5-glia-γ2, DQ2.5-glia-γ4b, DQ8-glut-H1 and DQ2.2-glut-L2 detected in the Osborne fractions were degraded during the gastroduodenal digestion; please refer to Table S3 for further details.

Finally, we focused our attention on the list of epitopes that survived the GD digestion process in light of their differential expression within the set of samples under investigation. Due to their resistance to proteolysis these sequences may pose a real risk to CD patients upon ingestion. The peptides encrypting the core epitopes DQ2.5-glia-α2, DQ2.5-glia-γ5, DQ2.5-glia-γ4c, DQ8-glia-γ1a, DQ2.2-glut-L1, together with other known epitopes that were not labeled with the standard nomenclature (ID numbers 227, 236, 542, 648, 717, 719, 923, and 1044 in Table S3), were detected in the GD digests and were expressed in all the five selected genotypes and in the reference semolina, as well. More interesting, the short-list displayed in Table 4 allowed discrimination of the analyzed set of grains, because it included only the intact epitopes that were not conserved in all samples. All the itemized epitopes were detected in the reference sample, whereas they were variably expressed in the five genotypes, as highlighted in Table 4. The analysis of the CD sub-types confirmed that genotype number 5 lacked most of the epitopic sequences listed in Table 4, as well as those belonging to α-, γ-, and ω-gliadins.

As already mentioned, we also assessed the immunogenic potential of the selected genotypes for wheat allergic patients. The general approach was very similar to what has already been described for celiac disease, except for a different bioinformatic tool exploited for epitope searches. The IEDB database (https://www.iedb.org/) was queried for linear epitopes recognition setting the specific constraints detailed in Section 2.7. The shortest linear epitope included in the database is 5AA long; therefore, we used this threshold for filtering the list of detected peptides. With this constraint, more than two thousand sequences were screened for each genotype (see Table 3 line 3). 

The epitope search carried out on Osborne fractions disclosed the occurrence of several epitopes belonging to both the albumin/globulin and storage proteins with a relative distribution displayed in Figure 4c. Only a limited percentage of the immunogenic sequences (2.8%) were assigned to the water soluble proteins, namely, α-amylase inhibitors (Tri a 15, Tri a 28), non-specific lipid transfer proteins (Tri a 14) and α-purothionin (Tri a 37), whereas most of the sequences encoding linear WA epitopes belonged to storage proteins, mainly LMW-GSs (Tri a 36) and ω-gliadins (Tri a 19), followed by HMW-GSs (Tri a 26), γ-gliadins (Tri a 20) and α-gliadins (Tri a 21). For the full list of detected WA epitopes and their occurrence in each genotype, we refer to Table S4 in the Supplementary Material.

Finally, the search for linear epitopes relevant for WA was also carried out on gastroduodenal digests, in order to investigate their susceptibility to the hydrolysis. For this in silico analysis, we valued the option “substring” provided by the portal for bioinformatics search, which made it possible to detect not only intact epitopic sequences, but also partial sequence matches, accounting for potential proteolytic degradation. The alignment accomplished in Table S4 made it possible to trace for all genotypes the fate of expressed epitopic sequences detected in Osborne fractions upon gastroduodenal digestion, disclosing which of them resisted the enzymatic cleavage and which were either partially or totally hydrolyzed, thus losing their immunogenic potential.

In line 4 of Table 3, we report the count of the hazard sequences (intact epitopes only) for WA patients scouted in the GD-resistant peptides list. Interestingly, also in this case, some differences between the reference semolina sample and the five selected genotypes were disclosed, particularly for genotype number 5 (PI56263), which was actually the most promising, with only the 40% of hazard peptides detected. Looking at the distribution of the sequences encrypting WA epitopes among different proteins, we found that the albumin/globulin immunogenic sequences were not detectable in GD digests (see Figure 4d), either as intact sequences or as hydrolyzed fragments; thus, we can conclude that they were completely hydrolyzed by digestive enzymes at the end of the duodenal phase. All the concerning full-length sequences were assigned to storage proteins with a higher prevalence of γ- and ω-gliadins, which often shared the epitopic sequences. As for the specific trend of each genotype, the histogram reported in Figure 5b presented a homogeneous reduction of immunogenic sequences among all classes of storage proteins. In Table S4, we also discriminated the epitopes sub-types with conserved amino acid sequences among the different wheat varieties. Several WA epitopes resistant to GD enzymes were expressed in the whole set of samples (ID numbers 17333, 38950, 49082, 49087, 49097, 51922, 52028, 52097, 52124, 113686, 113755, 113778, 113779, 148605, 148711, 148719, 148737, 148807, 148844, 148845, 173980, 174249, 174250, 192579, and 431006 in Table S4). The short-list reported in Table 5 is excerpted from Table S4, displaying only the intact epitopes that were not conserved and which may account for the differential immunogenic potential of the five genotypes.

## 4. Conclusions

In this investigation, we presented an in-depth analysis of the proteomic profile of selected durum wheat genotypes. An advanced proteomic approach based on high-resolution mass spectrometry was designed, which combined proteins/peptides sequence information retrieved by specific enzymatic digestions of Osborne protein fractions with protein digestibility information provided by in vitro simulated human gastroduodenal digestion experiments. This comprehensive approach not only made it possible to characterize the wheat genotypes and their genetic diversity, but also highlighted that the complexity of the model used to simulate in vitro the enzymatic digestion may influence the protein profile detected. The in silico evaluation of potential toxicity/immunogenicity for CD and WA patients proved that the selected genotypes encrypted a lower number of epitopes for both wheat related disorders, with particular attention towards the genotype number 5. Even if none of them can be considered safe for CD patients, grain with reduced amount of major T-cell stimulatory epitopes may help in the prevention of CD, since previous studies demonstrated that the amount and duration to gluten exposure are strictly linked to the initiation of this pathology [29,30]. As a future perspective of this work, the stability of protein expression and the consistency of the general trends observed here will be assessed with respect to different harvest years to confirm the relevance of the selected genotypes.

## Figures and Tables

**Table 1:** Details of the selected wheat genotypes (C: cultivated, NC: non-cultivated).

Sample Code	Accession	Taxonomic Classification	Year of Release	C/NC	Origin
1	Duetto	*T. turgidum ssp. durum*	2002	C	Italy
2	Colosseo	*T. turgidum ssp. durum*	1995	C	Italy
3	Lloyd	*T. turgidum ssp. durum*	1983	C	United States
4	Neolatino	*T. turgidum ssp. durum*	2007	C	Italy
5	PI 56263	*T. turgidum ssp. turgidum*	-	NC	Portugal, Lisboa

**Table 2:** Summary of the main features obtained by bottom-up proteomic characterization of the protein fraction collected for each genotype. # number of identified proteins or peptides.

Description	Fractions	Features	1	2	3	4	5
Single enzyme digests (CHY)	*F1+F2+F3*	*# Proteins*	359	373	339	395	276
		*# Peptides*	672	693	680	747	590
Multiconsensus of Single + Dual enzyme digests (CHY + THE/CHY)	*F1+F2+F3*	*# Proteins*	420	411	413	438	361
		*# Peptides*	719	777	767	814	698

**Table 3:** Summary of the GD-resistant peptides identified at the end of the duodenal phase and counting of the peptides encrypting full length epitopes relevant for celiac disease (CD) and wheat allergy (WA).

Description		REF	1	2	3	4	5
Total No. of identified peptides ^1^ (sequence length cut-off 9AA)	*Abs*	2027	1578	1732	1554	1513	1365
No. peptides containing intact epitopes relevant for CD	*Abs*	118	69	87	53	60	32
	*Rel*	100%	58%	74%	45%	51%	27%
Total No. of identified peptides ^1^ (sequence length cut-off 5AA)	*Abs*	2955	2469	2629	2440	2354	2136
No. peptides containing intact epitopes relevant for WA	*Abs*	208	133	165	121	136	86
	*Rel*	100%	64%	79%	58%	65%	41%

^1^ Filters applied for high confidence in identification like detailed in Section 2.6.

**Table 4:** Restricted list of gastroduodenal resistant CD epitopes highlighted for their differential expression among the analyzed set of samples (* source http://www.allergenonline.org/celiachome.shtml, accessed in June 2019).

CD Epitope Sequence (ID Number) *	HLA-DQ Molecules *	Protein *	9AA Restricted Core Epitope	Sample					
				REF	1	2	3	4	5
PQPQPFPSQQPY (7)	HLA-DR	α-gliadin	-	X	X	X	X	X	
QPFPQPQLPY (42)	DQ2	α-gliadin	DQ2.5-glia-α1a	X	X	X		X	
PFPQPQLPYPQ (45)	DQ2	α-gliadin	DQ2.5-glia-α1a DQ2.5-glia-α2	X		X			
PFPQPQLPY (53)	DQ2.5	α-9 gliadin	DQ2.5-glia-α1a	X	X	X		X	
PQPQLPYPQPQL (64)	DQ2	α-gliadin	DQ2.5-glia-α2	X		X			
FRPQQPYPQ (93)	DQ2.5	α-20 gliadin	DQ2.5-glia-α3	X	X	X	X	X	
PQQPYPQPQPQ (138)	DQ2	α-gliadin	-	X					
LGQQQPFPPQQPYPQPQ (151)	DQ2 (α1 * 0501, α1 * 0201)	α-gliadin	-	X	X	X	X		
LGQQQPFPPQQPY (152)	HLA-DR	α-gliadin	-	X	X	X	X		
QPFPQPQLPYSQ (164)	DQ2	α-gliadin	DQ2.5-glia-α1a	X	X	X		X	
PFPQPQLPYSQ (166)	DQ2	α-gliadin	DQ2.5-glia-α1a	X	X	X		X	
QPQPFLPQLPYPQP (185)	DQ2	α-gliadin	-	X		X			
PQPFLPQLPYPQ (187)	DQ2	α-gliadin	-	X	X	X	X	X	
PLQPQQPFPQQPQQPFPQPQ (224)	DQ2	ω-gliadin	DQ2.5-glia-γ4c DQ2.5-glia-γ5 DQ8-glia-γ1a	X					
PQQPQQPFPLQPQQPFPQQP (235)	DQ8	ω-gliadin	-	X					
QPFPLQPQQPVPQQPQ (976)	DQ2	ω-gliadin	-	X	X	X	X		
PFPQPQQPF (867)	DQ2.5	hor-1	DQ2.5-glia-ω1 DQ2.5-hor-1 DQ2.5-sec-1	X	X	X	X	X	
PQPQQPFPQ (891)	DQ2.5	hor-2	DQ2.5-hor-2 DQ2.5-sec-2	X	X	X	X	X	
PQQPFPQPQQPFPQ (911)	DQ2	ω-gliadin	DQ2.5-glia-ω1 DQ2.5-hor-1 DQ2.5-hor-2 DQ2.5-sec-1 DQ2.5-sec-2	X	X	X	X	X	
PQTQQPQQPFPQ (926)	DQ2	γ-gliadin	DQ2.5-glia-γ4c DQ8-glia-γ1a	X	X	X	X	X	
QSIPQPQQPFPQ (930)	DQ2	γ-gliadin	DQ2.5-hor-2 DQ2.5-sec-2	X	X	X		X	
QPFPQPQQPFPQ (935)	DQ2	γ-gliadin	DQ2.5-glia-ω1 DQ2.5-hor-1 DQ2.5-hor-2 DQ2.5-sec-1 DQ2.5-sec-2	X	X	X	X	X	
QQPQQPYPQ (458)	DQ2.5/DQ8	γ-1 and γ-5 gliadin	DQ2.5-glia-γ3 DQ8-glia-γ1b	X	X	X			
QQPYPQQPQ (464)	DQ2	γ-gliadin	-	X		X			
PYPQQPQQP (468)	DQ2	γ-gliadin	-	X		X			
PFPQPQQTFPQQPQLPFPQQ (502)	DQ2, DQ8	γ1-gliadin	-	X	X	X		X	
PFPQPQQTFPQ (503)	DQ2	γ1-gliadin	-	X	X	X		X	
PQQTFPQQPQLP (504)	DQ2	γ1-gliadin	-	X	X	X		X	
TQQPQQPFPQP (534)	DQ2	γ-gliadin	DQ2.5-glia-γ4c DQ8-glia-γ1a	X	X	X	X		
PFPQTQQPQQPFPQ (553)	DQ8 (DQ2/8)	γ-gliadin	DQ2.5-glia-γ4c DQ8-glia-γ1a	X	X	X	X	X	
PFPQPQQPQQPFPQ (644)	DQ8 (DQ2/8)	γ-gliadin	DQ2.5-glia-γ4c DQ8-glia-γ1a	X					
QPFPQLQQPQQP (650)	DQ2	γ-gliadin	-	X					
QQPPFSQQQQPVLPQ (701)	DQ2	γ-gliadin or LMW glutenin	DQ2.2-glut-L1	X	X	X	X		X
QQPPFSQQQQPQFSQ (751)	DQ2	LMW glutenin	-	X	X	X	X		X

**Table 5:** Restricted list of gastroduodenal resistant WA epitopes highlighted for their differential expression among the analyzed set of samples (* source https://www.iedb.org/, accessed in May 2019).

Protein (Allergen Code)	WA Epitope Sequence (ID Number *)	Samples					
		REF	1	2	3	4	5
α-gliadin (Tri a 21)	YLQLQPFPQP (148993)	X	X	X	X	X	
γ-gliadin (Tri a 20)	PQQPFPQLQQ (148736)	X	X	X	X	X	
γ-gliadin (Tri a 20)	QQQLPQPQQP (148860)	X			X	X	X
γ-gliadin (Tri a 20)	QQPVPQPHQPFSQQ (192607)	X					
ω-gliadin (Tri a 19)	QQPQQPFPLQ (52105)	X	X	X	X	X	
ω-gliadin (Tri a 19)	FPQQQFPQQQ (148610)	X		X			
ω-gliadin (Tri a 19)	PFPQPQQPFP (148713)	X	X	X	X	X	
ω-gliadin (Tri a 19)	PQQSPEQQQF (148748)	X					
ω-gliadin (Tri a 19)	QFPQQQFPQQ (148762)	X		X			
ω-gliadin (Tri a 19)	QQFPQQQFPQ (148828)	X		X		X	
ω-gliadin (Tri a 19)	QQLQQPFPLQ (148834)	X		X	X	X	
ω-gliadin (Tri a 19)	QQPIPVQPQQ (148841)	X	X	X	X	X	
ω-gliadin (Tri a 19)	QQPQQPFPQL (148843)	X					
ω-gliadin (Tri a 19)	QQQFPQQPPQ (148856)	X		X			
ω-gliadin (Tri a 19)	QQQFPQQQFP (148857)	X		X		X	
ω-gliadin (Tri a 19)	QSPEQQQFPQ (148898)	X					
ω-gliadin (Tri a 19)	FPQQQFPQQQFPQ (173928)	X		X			
ω-gliadin (Tri a 19)	PQQQFPQQQQFPQ (173960)			X			
ω-gliadin (Tri a 19)	PQQSPEQQQFPQQ (173967)	X					
ω-gliadin (Tri a 19)	QQQFPQQQFPQQP (173992)	X		X			
ω-gliadin (Tri a 19)	EQQQFPQQQF (190740)	X					
ω-gliadin (Tri a 19)	PQQPQQFPQQ (190898)	X		X		X	
ω-gliadin (Tri a 19)	PQQQQFPQQQ (190900)	X	X	X	X		X
ω-gliadin (Tri a 19)	QPQQFPQQQF (190924)	X				X	
ω-gliadin (Tri a 19)	QQFPQQQQFP (190931)	X	X	X	X		X
ω-gliadin (Tri a 19)	QQFPQQQQLP (190932)		X	X			
ω-gliadin (Tri a 19)/γ-gliadin (Tri a 20)	QTQQPQQPFP (52600)	X	X	X	X	X	
HMW-glutenin (Tri a 26)	QPGQGQQGQQPGQG (113765)	X	X	X		X	X
HMW-glutenin (Tri a 26)	QPGQGQQPGQGQPG (113766)			X			
HMW-glutenin (Tri a 26)	QQPGQGQQPGQGQQ (113781)	X					

## Supplementary Materials

The following are available online at https://www.mdpi.com/2072-6643/11/10/2321/s1, Figure S1: Comparison of the total protein content assayed in all protein fractions collected for each genotype. Figure S2: SDS-PAGE of the three protein fractions F1 (a), F2 (b), F3 (c) for all five wheat genotypes. Fractions F1 and F2 were separated on a 4-20% gradient gel, whereas the fraction F3 was separated on a 12% gel. Table S1: Full list of proteins and peptides identified in Osborne protein fractions (multiconsensus of peptide pools obtained with CHY and THE/CHY); Table S2: Full list of GD resistant peptides; Table S3: Full list of CD epitopes detected and their distribution among different genotypes and set of samples. Table S4: Full list of WA epitopes detected and their distribution among different genotypes and sets of samples.
